# NN-Based 8FSK Demodulator for the Covert Channel

**DOI:** 10.3390/s22197181

**Published:** 2022-09-21

**Authors:** Krystian Grzesiak, Zbigniew Piotrowski

**Affiliations:** Institute of Communications Systems, Faculty of Electronics, Military University of Technology, 00-908 Warsaw, Poland

**Keywords:** wireless communication, co vert channel, dirty constellation, steganography, LPD, security, neural network

## Abstract

In this article, a superposition-based covert channel and its demodulator were proposed and examined. As a covert waveform, an 8FSK modulation was selected. The impact of the channel estimation error and resulting imperfect SIC operation (successive interference cancelation) on the covert information demodulation process was considered. Especially for this imperfection, an NN-based demodulator was proposed. The superiority of this solution over the traditional 8FSK correlator-based receiver was examined for various cases, including the hard- and soft-decision detectors. It was proven that, although NN does not provide BER values equal to zero, even for the perfect SIC, it generally overcomes the traditional correlator-based 8FSK demodulator. Simulation results showed that the NN-base demodulator, in the case of additional covert channel coding, provides error-free demodulation, even for four-times greater channel gain error.

## 1. Introduction

The first use of the term “covert channel” occurred in research study [1]. It focused on the exchange of data between programs. It defined the covert channel as a communication channel that is not at all designed or intended for the transmission of information. Nowadays, it is assumed that any manner of communication that violates the system security policy is a covert channel. In the physical layer of wireless communication, there were proposed solutions [2,3,4,5], based on the so-called post-modulation [6] method, which embeds the hidden data stream after the modulation stage. In this method, the covert modulated signal is superposed on the cover (unhidden) signal. The hidden signal must be much weaker than the cover signal to avoid detection (in the sense of the low probability of detection). Moreover, additional phase shift mechanisms [2] can be implemented to decrease detection through statistical analysis.

The previous studies on covert channels consider only selected, coherent types of modulation for hidden signals. Generally, it was assumed that the separation of the superimposed signals (SIC operation) can be performed in a perfect way, which means that the receiver has perfect information about the cover signal. It is far from practice and reality. In this paper, we analyze an 8FSK modulation superimposed onto a QAM signal (cover). For the so-defined covert channel, an NN-based demodulator was proposed. To support the examinations, we compared the presented demodulator with a traditional FSK correlator-based demodulator. Our results indicate that the NN-based demodulator outperforms 8FSK correlator-based demodulator for imperfect SIC measured by channel gain estimator error.

In this paper, we present a strictly defined problem connected with a covert channel that combines several topics, such as steganography, FSK demodulation, impartments in the receiver, soft/hard metrics, and neural networks, as well as their applications. So, it needs to be analysed and merged with the use of several literature references to enhance the physical secrecy and quality of the transmission.

The idea of the use of a neural network in the demodulation process and, generally, in the physical layer of wireless communication [7] is not quite new. A NN-based BPSK demodulation is a subject of the article [8]. A fully convolutional neural network with variable input and output length, called DemodNet, to demodulate BPSK and QAM modulation was presented in [9]. DemodNet provides, under the AWGN channel, results that are very close to the traditional methods. What is important in this approach is the soft and hard demodulation realized by NN. To reduce the overall computational complexity of the soft-decision, a trainable universal neural network-based demodulator architecture was introduced in [10] for QAM signals

In the literature, much less attention has been devoted to FSK demodulators, when it comes to soft demodulation and neural networks, in general. A soft-decision Viterbi decoder for FSK demodulation was presented in [11]. However, it deals with frequency discriminator for FSK demodulation. Soft-decision metrics for FSK signalling, in the presence of Cauchy and Gaussian noise, are derived in [12]. The authors proposed utilizing correlators in the detection process, which is useful for dealing with non-coherent demodulation. A neural network, as a technique for BFSK demodulation, was presented in [13]. The authors simulated the demodulation process over the AWGN Rayleigh fading channel, but the NN works in a hard-decision manner.

The problem of the imperfect SIC operation (caused by channel gain estimation error) and superposition of signals are closely related to NOMA, specifically P-NOMA (power non-orthogonal multiple access) systems. In the case of NOMA, SIC errors have less impact on the system performance. There are some positions in the subject literature [14,15].

In summary, to the best of the authors’ knowledge, there are neither studies on high or-dered FSK modulation as a covert modulation nor comprehensive study regarding its non-perfect demodulation (in a presence of non-perfect SIC). Therefore, the purpose of this article is to fill this gap, as follows:

- To ascertain the pros and cons of using 8FSK modulation we have compared the performances of the main modulation schemes used to create a covert channel;
- We examined the imperfect SIC operation on 8FSK demodulation;
- We proposed both soft- and hard-decision NN-based demodulator for covert channel and compared it with correlator-based FSK detector.

The rest of the paper is organized as follows. Section 2 briefly presents the covert channel model and receiver based on SIC operation. This section also justifies the use of 8FSK as a covert modulation. Section 3 presents sources of imperfect SIC and its impact on covert modulation reception. Section 4 presents the NN-based 8FSK demodulator and test scheme used for its evaluation. Section 5 presents the simulation results for the NN-based 8FSK demodulator and compares it with the traditional correlator-based 8FSK demodulator. Finally, the conclusions are presented in Section 6.

## 2. Model of the Covert Transmission

### 2.1. Covert Transmission Model

**Figure 1:**
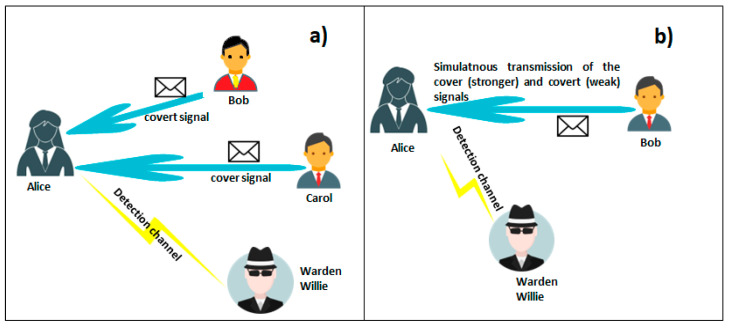
Scenarios for covert channel: (**a**) general model; (**b**) simplified model.

The covert transmission model tends to assume the existence of a standard cast of characters: Alice, Carol, Bob, and Willie [16]. In this model, it is considered a scenario where that Bob wants to send information to Alice, in the presence of Carol’s transmission (Figure 1a). The steganalyser is represented by the Warden (Willy). His knowledge of the cover transmission (waveform, variation, statistical properties, etc.) determines the possibility of the detection, where the covert channel transmission takes place. If there is no knowledge regarding covert transmission, Willy has no chance, in the sense of LPD (low probability of detection), to detect Bob. In other words, the covert message can be detected only when the non-covert message, where the covert message is superimposed, can be decoded [17]. Model Figure 1a can be simplified to the one presented in Figure 1b by assuming that Bob simultaneously transmits (but not necessarily synchronously) the cover and covert signals. In this case, Bob perfectly controls the cover and covert powers. In this study, for simplicity, we assume the above model.

### 2.2. Selection of the Modulation for Hidden Transmission

Bob’s ability (from Figure 1) to hide the fact of communication mainly depends on his ability to make covert signals look like noise. Therefore, the power of the covert signal should be kept as small as possible; in order to avoid detection by the outsider (Willie) [18].

Moreover, a covert waveform can be modified to impede steganalysis. So far, in the case of typical coherent transmission (QAM/PSK modulation), the covertness was increased by constellation point rotations [2,3]. The impact of the shape of the covert con-stellation points and resulting IQ histograms will be discussed in the next section.

It is also worth considering receiver problems. From the demodulator point of view, non-coherent demodulators are simpler. This issue arises because the carrier phase can be altered by the channel and has an impact on the accuracy of the detection. If there is not a perfect phase coherence, the non-coherent demodulation constitutes a good solution and can make it easier to convert channel demodulation. In addition to the foregoing, it is worth noting that, in contrast to the typical communication model, a covert channel generally does not require a high data rate. The low variance of the covert signal is compensated by the increased duration of the symbol.

These scenarios and assumptions lead us to the main assumptions for covert signal modulation:

- No constant constellation points (higher immunity to steganalysis [2,3,4]);
- Non-coherent detection (easier detection, lack of expensive, and complex recovery circuit);
- Possible low BER (bit error rate) for given energy per symbol to noise power (reliable transmission).

**Figure 2:**
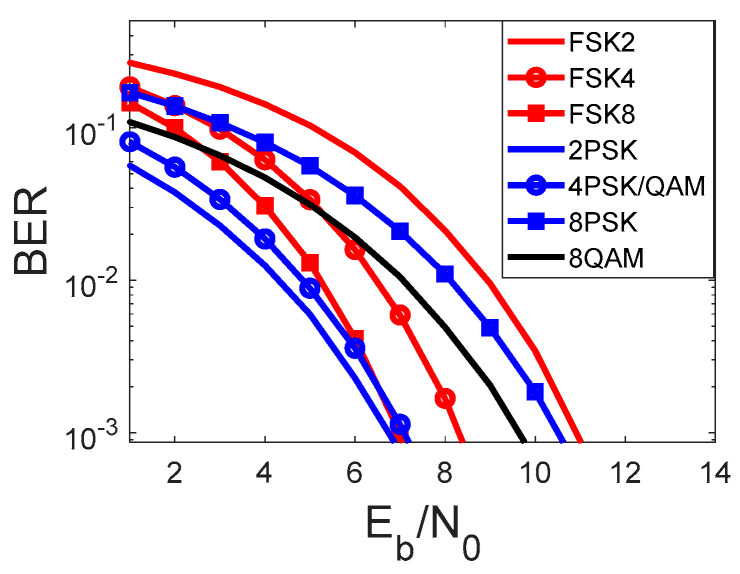
BER curve vs. $E_{b}/N_{0}$ for FSK, PSK, and QAM modulation.

FSK modulation fulfills the first two of three criteria. The last criterion must be carefully analyzed. The best way to do this is as follows: BER curves are used for illustration of the modulation performance. Figure 2 shows the BER performance for coherent (QAM and PSK) and non-coherent demodulation (FSK), taking the different modulation orders into consideration.

The performance analysis of the two-ordered modulation under the minimum required $E_{b}/N_{0}$rate shows the superiority of 2PSK modulation. The 2FSK modulation required 4 dB more energy per bit. By increasing the order of the PSK/QAM modulation, the higher $E_{b}$ that is required, the lower the bandwidth. In the case of FSK, the higher modulation order results in lower energy per bit and higher bandwidth. From a covert channel perspective, it is most important to choose a modulation scheme that has a lower required $E_{b}/N_{0}$ for the given BER. This justifies the use of 2PSK, 4PSK, and QAM modulation in the previous papers of the author [2,3,4,5].

As was shown in Figure 2, 8FSK provides similar results (in the sense of energy per bit), and that is why it became the waveform for the covert information and subject of our analysis.

### 2.3. The System Model for Cover Communication

**Figure 3:**
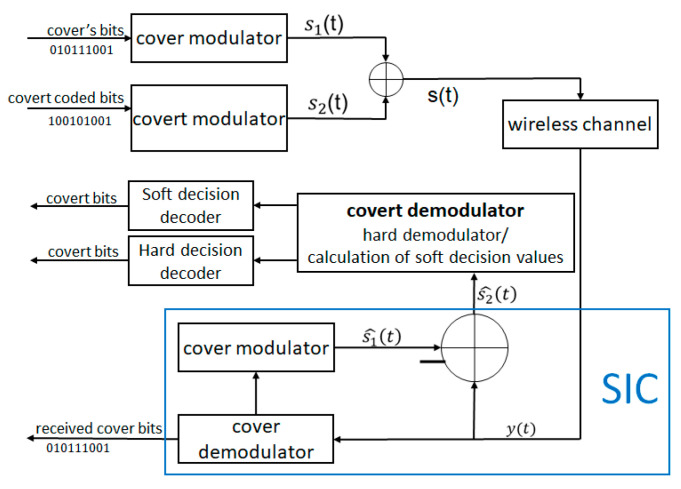
Transmitter/receiver structure for covert channel.

Figure 3 shows in a detail the system model (compatible with Figure 1b) used to describe principles of a covert channel, based on the superposition of signals. The cover and covert signals are transmitted simultaneously (in our case, not necessarily synchronously). To increase the BER performance, and for better analysis of our proposed solutions, we assumed channel coding before covert data transmission. The coding was not carried out for cover bits. Next, the superimposed signals with different power levels were transmitted via a wireless channel. In the receiver, SIC operation (successive interference cancellation) was performed to recover the covert signal. SIC, in short, means subtraction operation, where the minuend represents the received combined signal (superposition of cover and covert modulated signals), and the subtrahend is the recovered signal of the cover (after remodulation).

The transmitter signal is shown in Equation (1) [14].

(1) $$s\left(t\right)=s_{1}\left(t\right)+s_{2}\left(t\right),$$

where $s_{1}\left(t\right)$ is the signal of the cover (with the variance $\sigma_{1}^{2}$), and $s_{2}\left(t\right)$ is the covert signal (with the variance $\sigma_{2}^{2}$). To ensure the secrecy of communication, the variance of the signals should satisfy the inequality $\sigma_{2}^{2}<<\sigma_{1}^{2}$. If the receiver (user) is aware that additional information is embedded, it is able to decode (demodulate) the covert message. This is achieved by removing the cover signal from the received signal. An uninformed user treats signal $s\left(t\right)$ as a noised version of $s_{1}\left(t\right)$.

The received signal is given as Equation (2) [15]:

(2) $$y\left(t\right)=h\cdot s\left(t\right)+n=\sum_{i=1}^{2}h_{i}\cdot s_{i}\left(t\right)+n.$$

In this model, h represents the channel gain (e.g., Rayleigh fading channel) between the transmitter and receiver, and it is represented by a circularly-symmetric, complex Gaussian random variable. The symbol n represents Gaussian noise.

Perfect SIC operation means that the recovery and re-modulation of the signal $s_{1}\left(t\right)$ are achieved without any errors, which means [15], as shown in Equation (3):

(3) $${\hat{s}}_{2}\left(t\right)=y\left(t\right)-{\hat{s}}_{1}\left(t\right)=y\left(t\right)-s_{1}\left(t\right)=s_{2}\left(t\right).$$

Assumed that a perfect SIC operation can be performed, the simulation was conducted for the following parameters.

**Figure 4:**
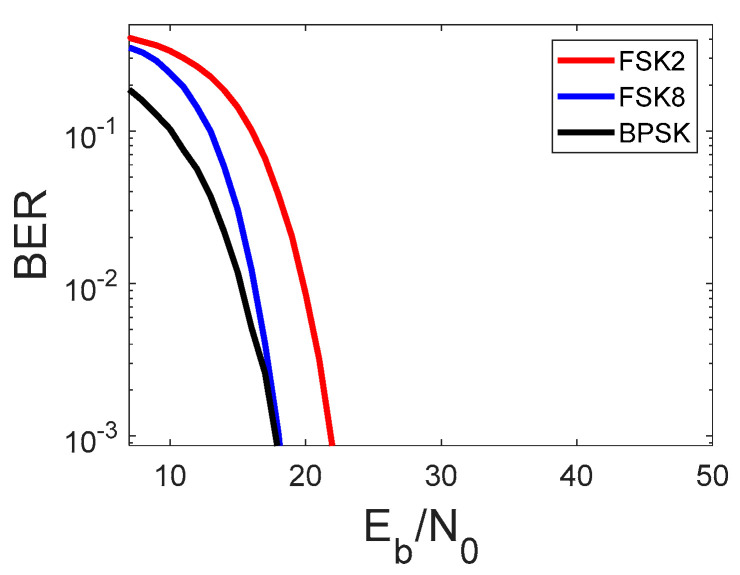
The comparative simulation of the covert data transmission in AWGN channel.

Different numbers of samples per symbol for different covert modulation (Table 1) were adopted to achieve the same covert data rate. The results of the simulation are depicted in Figure 4, where the value $E_{b}/N_{0}$ refers only to the cover signal (16QAM).

**Figure 5:**
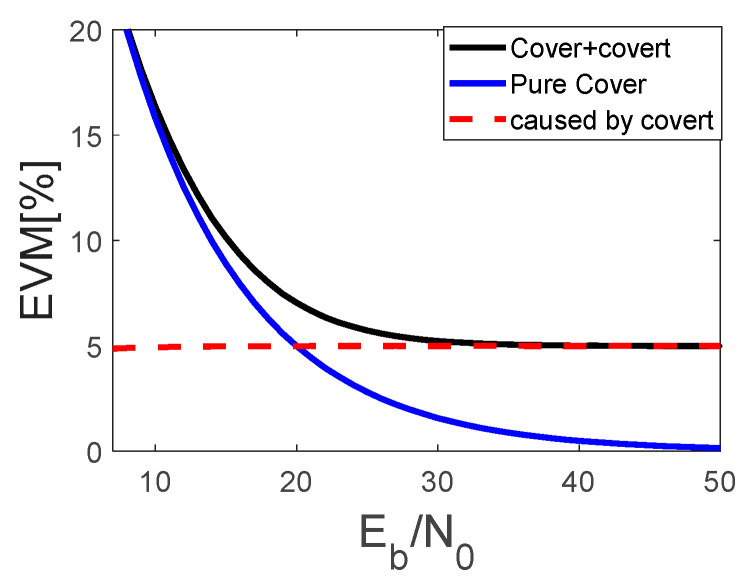
EVM introduced by covert signal on the cover signal constellation.

**Figure 6:**
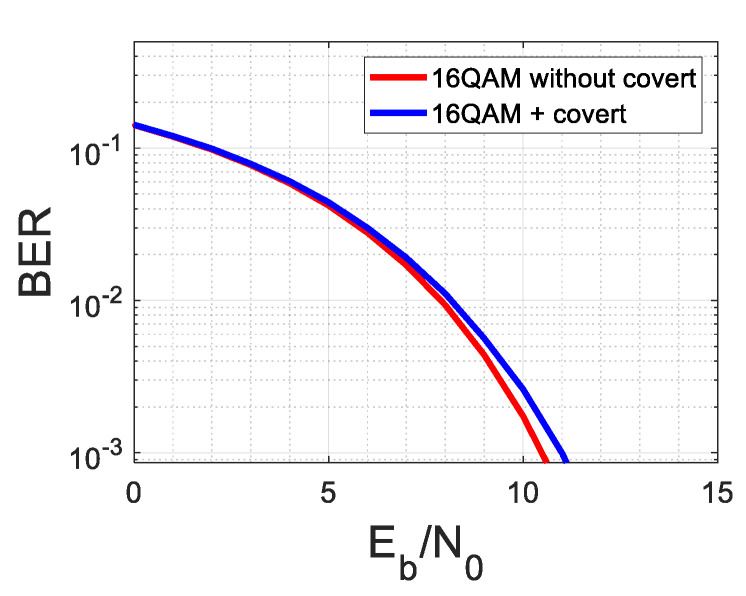
Comparison on BERs for 16QAM and 16QAM disturbed by covert signal in AWGN channel.

The covert signal causes additional distortions and degrades the system performance that can be measured by EVM. For this case, the results of the simulation are depicted in Figure 5 and Figure 6.

From the results depicted above, we can conclude that FSK, 8FSK, and BPSK have the same impact on the cover signal. Slightly worse transmission parameters of 8FSK in relation to BPSK (Figure 4) may be treated as a cost of the non-coherent demodulation.

There remains the question of the steganalysis mentioned in the previous section. It is usually based on histograms. It is achieved by carrying out the I/Q analysis of the received signal s(t) and examining its histograms.

**Figure 7:**
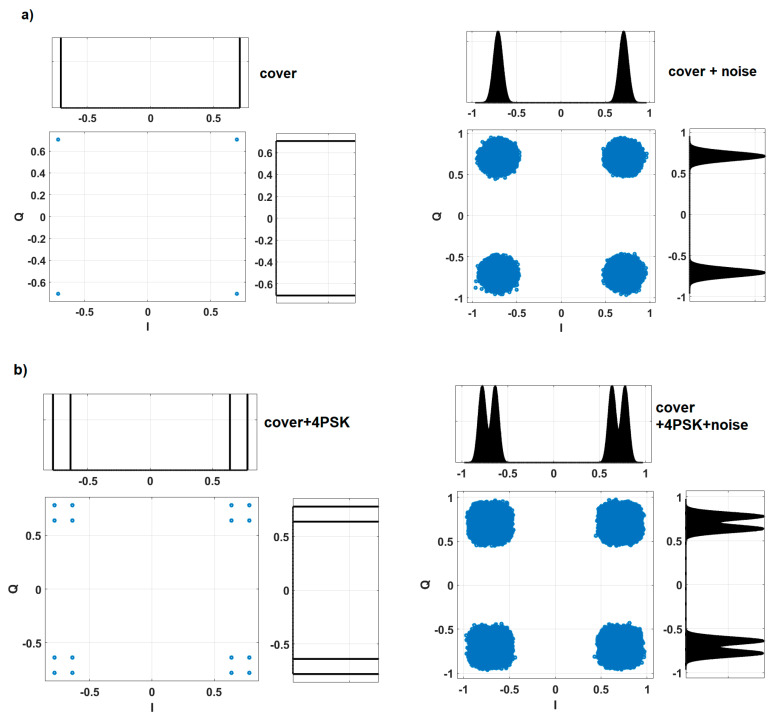

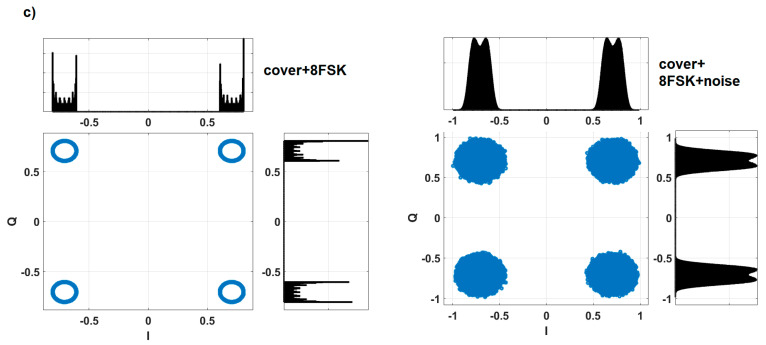
Constellations and histograms for (**a**) cover (QAM); (**b**) cover + 4PSK; (**c**) cover + 8FSK for noiseless and gaussian noise.

The example of histogram-based steganalysis is presented in Figure 7. For a clearer graphical presentation, we assumed QAM as the cover. We compare 8FSK IQ histograms with covert modulation 4PSK. The covert modulation is 4PSK taken for the analysis result from comparable bit energy for the given BER (presented in Figure 2).

As shown in Figure 7, adding a hidden signal to the cover signal inevitably changes the properties of the second one. For the countermeasure for covert channels, steganalysis looks for these changes. When the warden (Figure 1) has perfect knowledge about the cover signal (plus channel gains, noise, and other disturbances), it is not possible to ensure covertness (Figure 7). Increased resistance 8FSK to steganalysis comes from the fact that IQ histograms (estimation of the probability density functions) are more similar to the cover histograms than 4PSK, especially in the case of the noisy channel (Figure 7b vs Figure 7c). Basically, coherent modulation (like N-order phase modulation) always provide a limited number of states on IQ constellation and, thus, bins on IQ histograms. In contrast, after observation of the N-FSK signal, the obtained histograms have many more bins, in a specified range. That is why FSK modulation is less prone to steganalysis.

Graphical representation of the signal in histogram form is used to decide on the detection of hidden transmission. Numerical values of the similarity of the histograms can be calculated by Kolmogorov–Smirnov (KS) test [3,19].

## 3. Imperfect SIC Operation

**Figure 8:**
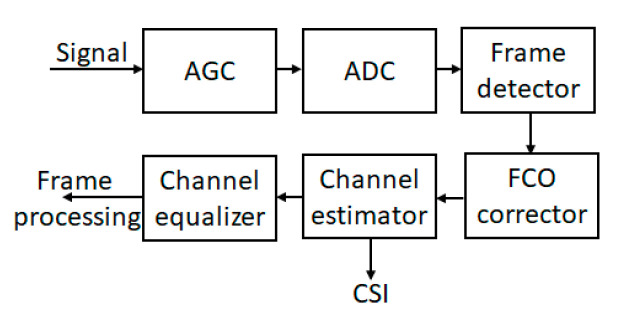
Typical receiver block diagram.

The severe amplitude and phase fluctuations inherent to wireless channels significantly degrade the BER performance of the signal. Moreover, errors in the demodulation process come from every part of the transmitter and receiver, including antenna systems [20,21,22,23] The typical receiver block diagram is depicted in Figure 8.

The main sources of distortion considered in our paper are AGC and CE (channel estimator). Other errors can come from ADC (analog to digital converter) and FCO (frequency carrier oscillator) corrector. The impact of the channel gain error and hardware impartments for SIC were presented in [24,25], whereas the mean-reverting signal strength was presented in [26]. AGC and CE errors lead to non-ideal SIC operation. As a result, SIC cancels only a part of the cover signal.

The covert signal after non-deal SIC is provided by [15], as shown in Equation (4):

(4) $${\hat{s}}_{2}\left(t\right)=\frac{\left(h-\hat{h}\right)s_{1}\left(t\right)}{\hat{h}}+\frac{h\cdot s_{2}\left(t\right)}{\hat{h}},$$

where $\hat{h}$ is a channel gain estimator and represents in our paper AGC and CE errors.

When the channel gain estimator is equal to zero, i.e., $e_{x}=\left|h\right|-\left|\hat{h}\right|$ = 0, then ${\hat{s}}_{2}\left(t\right)=s_{2}\left(t\right)$, that indicates perfect SIC. In the case of covert transmission, when $\sigma_{2}^{2}<<\sigma_{1}^{2}$, even a small error $e_{x}$ causes severe damage to the covert signal. In the subsequent discussion, the normalized channel gain estimator error will be used to evaluate the demodulator’s performance, provided by [27], as shown in Equation (5):

(5) $$e_{abs}=\frac{\left|h\right|-\left|\hat{h}\right|}{\left|h\right|}\cdot 100\%.$$

**Figure 9:**
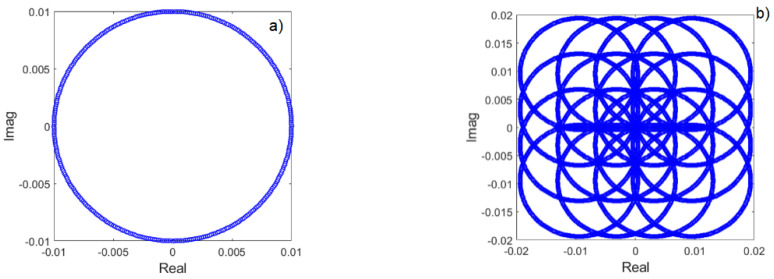
Constellation of the transmitted 8FSK covert signal after perfect (**a**) and imperfect (**b**) SIC operation ($e_{abs}$ = 2%). Variance of the cover $\sigma_{1}^{2}$ =1 and variance of covert $\sigma_{2}^{2}=0.01\sigma_{1}^{2}$.

The example of the IQ constellation of the covert signal after perfect ($e_{abs}$ = 0) and imperfect SIC ($e_{abs}$ = 2%) for 8FSK (covert) and 16QAM as a cover signal is depicted in Figure 9. However, such a graphical presentation does not reflect the detection performance, so the detailed assessment and numerical evaluation must be performed via simulation.

## 4. NN-Based 8FSK Demodulator and Its Evaluation

**Figure 10:**
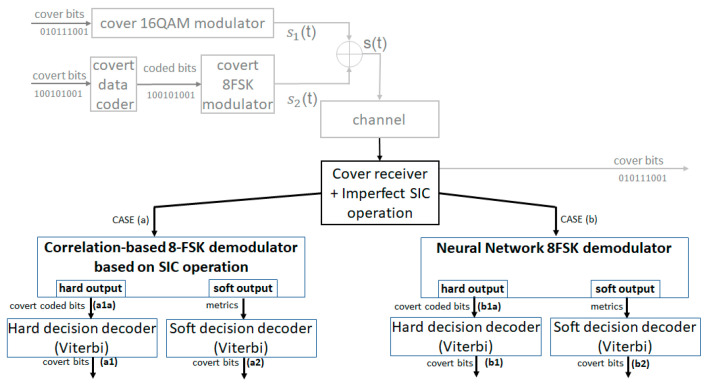
Block diagram of test scheme (all methods under test).

To evaluate the impact of channel gain estimation error on covert signal demodulation (consequently the imperfect SIC) we used a test scheme presented in Figure 10. This scheme shows that correlation-based (Figure 10, case (a)) and NN-based (Figure 10, case (b)) demodulators were tested under the same conditions, including hard and soft decoding.

Covert data are coded (Figure 10) and modulated using the 8FSK modulation. Next, the signal is superimposed onto the 16QAM cover signal. For simplicity, we assume that the wireless channel does not introduce any errors (noise-free channel). The cover signal is distorted only by the covert signal, which means, in our case (very low variance of the covert signal), its error-free demodulation. Then comes the main purpose of this paper: a comparison of the typical (correlator-based) 8FSK and NN-based demodulators, in the presence of the SIC error. They both realize hard- and soft-output demodulation. Evaluation of these methods was achieved using BER ratio vs. $e_{abs}$ for coded data (hard-output demodulation) and decoded data (hard- and soft-output demodulation). Every demodulation/decoding was made separately, with the use of the same input set. In our simulation, NN has been implemented in Keras with Tensorflow. Input data, as in all simulations reported here, were created in Matlab. In the next section, the main elements of our test will be described, especially the NN-based and correlator-based demodulators.

### 4.1. Correlator-Based 8FSK Demodulator

Demodulation can be achieved in either a soft or hard manner. Hard demodulation converts the received symbols into bit streams. Compared with hard demodulation, soft demodulation retains more channel information. Soft demodulation provides the level of confidence of each bit, according to the received symbols. The commonly used method of soft-decision is the estimation method, based on the logarithmic likelihood ratio (LLR). When the parameters of the channel and noise are unknown, the unknown parameters can be replaced by their maximum likelihood estimates (this leads to the so-called generalized likelihood ratio (GLR)). The output of the demodulator is then transferred to the channel decoder. As a result, it can improve the posterior probability of data decoding and reduce the bit error rate.

**Figure 11:**
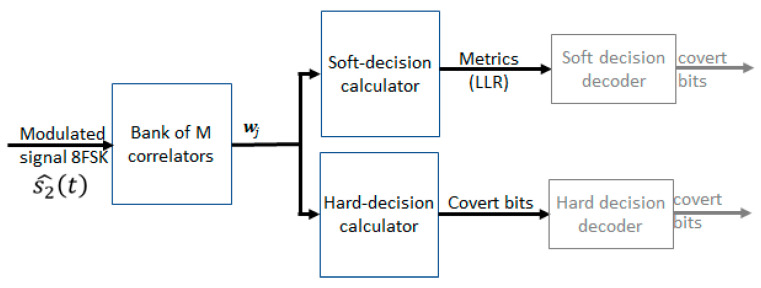
Block diagram of the 8FSK correlator-based hard and soft demodulator.

The block diagram of the 8FSK hard and soft demodulator is presented in Figure 11. The MFSK modulated signal $s_{2}\left(t\right)$ for the order M=8 is composed of eight elementary components [11], as shown in Equation (6):

(6) $$s_{2,j}\left(t\right)=\left[s_{2,1}\left(t\right),s_{2,2}\left(t\right),\ldots ,s_{2,j}\left(t\right)\right],j=1,\ldots ,M$$

Every elementary component is given by [28], as shown in Equation (7):

(7) $$s_{2,j}\left(t\right)=\exp(-j2\pi mf_{j}t)$$

and frequency separation equals $\Delta f_{0}$.

The output of the j−th correlator $w_{i}$ is given by [28], as shown in Equation (8):

(8) $$w_{j}=\left|\frac{1}{T}\int_{T}{\hat{s}}_{2}\left(t\right)\cdot conj\left(s_{2,j}\left(t\right)\right)dt\right|.$$

The hard-decision calculator determines the maximum value among all $w_{j}$; hence, the symbol value $c_{i}$ [12], as shown in Equation (9):

(9) $$c_{i}=max(w_{j}^{2}).$$

Each received symbol represents $log_{2}M$ coded bits. The soft-decision of coded symbols are generated by the demodulator, in the form of a log-likelihood ratio (LLR), and passed to the decoder. The LLR of the jth coded bit $b_{j}$ is defined as [12,28], as shown in Equation (10).

(10) $$L_{j}≜\log\left(\frac{\mathrm{Pr}[b_{j}=0|w]}{\mathrm{Pr}[b_{j}=1|w]}\right)$$

where w = [$w_{1},w_{2},\ldots ,w_{M}$] is a vector of the outputs of all correlators.

In our case, because of unknown channel parameters, we use GLR metrics given by Equation (11):

(11) $$L_{j}\left(w\right)=log\left(\frac{\sum_{i:b_{j}=1}{\left(\sum_{k=0,\;k\neq i}^{M-1}w_{k}^{2}\right)}^{-M}}{\sum_{i:b_{j}=0}{\left(\sum_{k=0,\;k\neq i}^{M-1}w_{k}^{2}\right)}^{-M}}\right)$$

### 4.2. NN-Based 8FSK Demodulator

**Figure 12:**
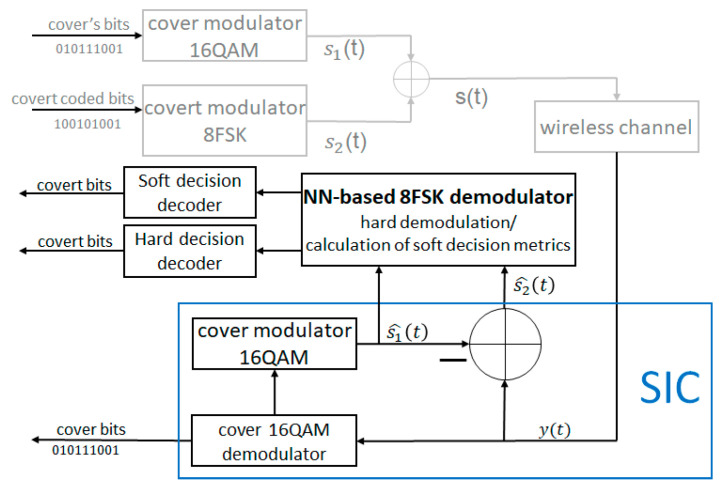
Block diagram of the receiver structure with the NN-based 8FSK hard and soft demodulator.

**Figure 13:**
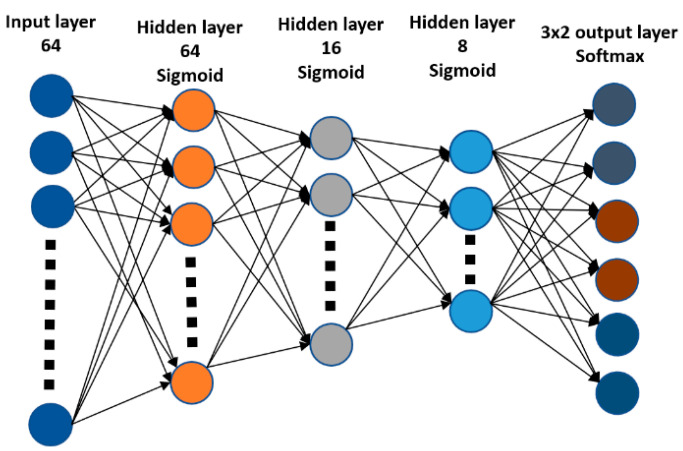
Deep Neural Network (DNN) architecture for covert channel demodulation.

The block diagram of the receiver with the NN-based FSK demodulator is presented in Figure 12, with the NN architecture in Figure 13. The NN-based covert channel demodulator is a result of searching for the optimal solution. Originally, it was assumed that NN can perform SIC operation and demodulation simultaneously. However, NN was not able to learn, due to the fact that the covert information contained in far decimal places was lost by it. Finally, the input vector is composed of signals ${\hat{s}}_{1}$ (signal after remodulation process, i.e., demapping) and ${\hat{s}}_{2}$.

It was assumed that every symbol of the 8FSK consists of 16 complex samples (16 real and 16 imaginary). Accordingly, for the input of the neural network (Figure 13), a vector of length 64 is supplied. There are three hidden layers with a sigmoid activation function. The output layer consists of three softmax activation functions, one for each bit. In the simplest case, such a neural network, it operates as a classifier that classifies the received signal into the corresponding transmitting bit combination. To train the NN, we use hard bit information as labels. The cross-entropy was used as the loss function during the training. The best performance of the model was achieved for the batch size, in each training round set to 256 and epochs to 1000. The input set consisted of 100,000 samples, where 80% of samples were used to train NN, with 20% for validation.

As mentioned before, the presented NN can work as both hard- and soft-decision demodulators. The hard-decision is based on the selection of the softmax output with a higher value. The decision is made for every 3 bits (data symbol). In the case of soft demodulation, we utilize the property, and that sigmoid output represents the probability that the bit has a certain value, 0 or 1. For every two outputs, for every softmax function (${\mathrm{out}}_{1,N}$ and ${\mathrm{out}}_{2,N}$, N=1, 2, 3), the relation can be expressed as: ${\mathrm{out}}_{1,N}=1-{\mathrm{out}}_{2,N}$. Thus, the soft-decision metrics are given as [9], as shown in Equation (12):

(12) $$\xi_{j}=log\frac{out_{1,N}}{out_{2,N}}=logit\left(out_{1,N}\right),N=1,2,3$$

### 4.3. Channel Coder

**Figure 14:**
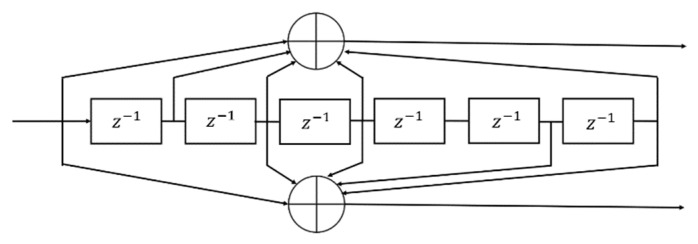
Encoder for rate R = ½, and constraint length K = 7 convolutional code.

In our simulation, for both demodulators, we implemented the standard convolutional encoder and Viterbi decoder [28] rate of ½ with generator polynomial $G=\left[171,\;133\right]$ for constraint length 7 (Figure 14).

## 5. Simulations Results

We have conducted a simulation to assess the BER performance in the presence of imperfect SIC operation measured by $e_{\mathrm{abs}}$ (distortion expressed in %). The main parameters of the simulation are shown in Table 2. It was assumed that $e_{\mathrm{abs}}$ is constant over the transmission of the one symbol 8FSK. The evaluation of the correlator and NN-based demodulator was done at different stages of the covert signal data reception process:

- Hard 8FSK demodulation;
- Hard/soft 8FSK demodulation with a covert data decoder.

### 5.1. Hard Demodulation (Without Channel Coder)

**Figure 15:**
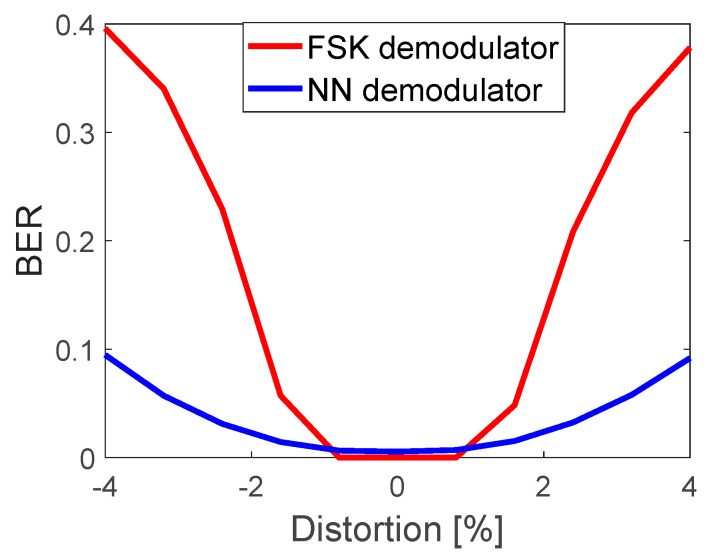
Hard-decision FSK correlator-based and NN-based demodulator. BER performance vs. $e_{abs}$[%].

Figure 15 shows the performance comparison of the 8FSK correlator-based and NN demodulators (output a1a vs output b1a, Figure 10). The advantage of the 8FSK demodulator, which is obvious, is perfect demodulation in the absence of channel estimation gain error. The NN-based demodulator has BER ≠ 0, even for $e_{abs}$ = 0. The superiority of the NN over the correlator-based detector appears for $e_{abs}$ > 1%. Imperfect SIC severely degrades FSK demodulator, with the growth $e_{abs}$, whereas NN considerably mitigates its effect. It can be assumed that channel coding can improve BER. The impact of channel coding is presented in the next section.

### 5.2. Demodulation with Hard/Soft Viterbi Decoder

As expected, the Viterbi decoder can improve BER performance, but the final results strongly depend on the demodulator.

**Figure 16:**
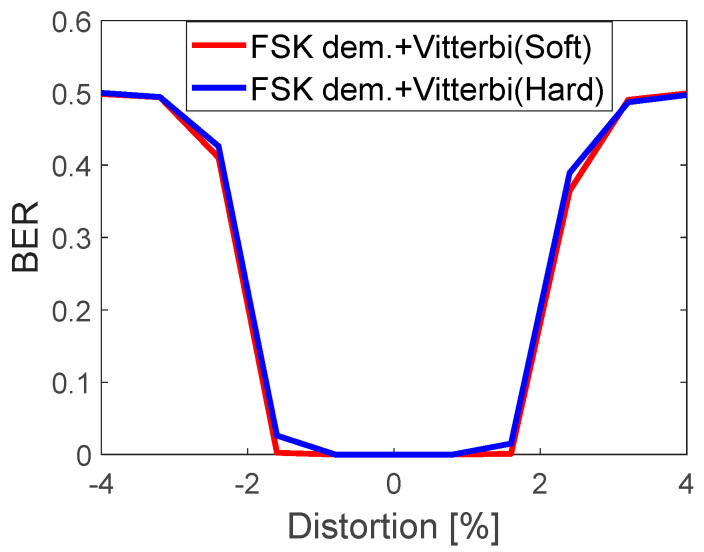
Hard and soft correlator-based FSK demodulator with Viterbi decoder. BER performance vs. $e_{abs}$[%].

Figure 16 shows the performance of the correlator-based 8FSK demodulator for hard and soft decoding (output a1 vs output a2, Figure 10). In his case, soft decoding has a negligible impact, in comparison to hard decoding. It is due to the fact that the metrics are calculated for unknown channel parameters.

**Figure 17:**
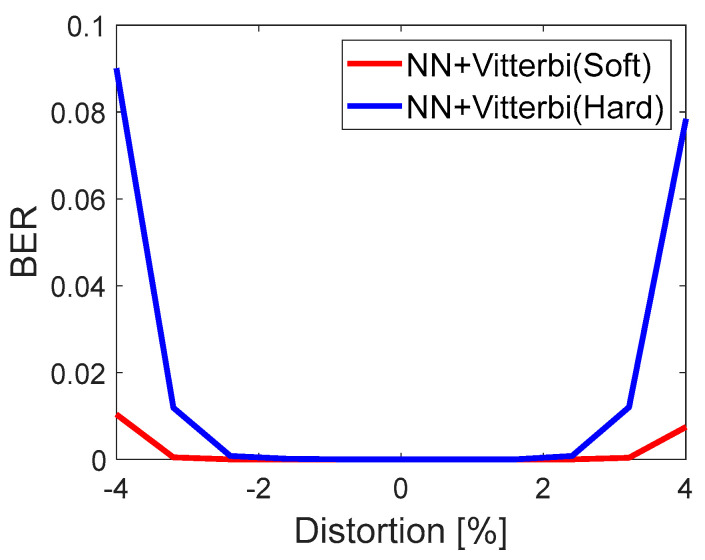
Hard and soft NN-based FSK demodulator with Viterbi decoder. BER performance vs. $e_{abs}$[%].

In the case of the NN demodulator (output b1 vs output b2, Figure 10), the channel coding significantly improves the performance of the system (Figure 17). It guarantees, in comparison to the system without a coder (Figure 15), the BER = 0 for $e_{abs}$ = 0. Moreover, the soft decoding, in addition to NN, remarkably enhances detection. This results from the fact that the metrics calculated based on NN perform much better than those that are theoretically calculated.

## 6. Conclusions

In this article, the 8FSK modulation was proposed for covert communication based on the superposition of the signal. This modulation provides the possibility of transmitter and receiver low complexity and assists considerably in the effective practical implementation of such a system. The main advantage of this solution is the non-coherent transmission and a lower probability of detection by the intruder, thanks to non-constant constellation points on the constellation diagram. This is a major difference from a typical solution, based on the constant point of the phase modulation. Inherently, the covert signal suffers from non-deal SIC operation caused by hardware impairments and estimation channel gain error, mainly because of the low energy of the hidden signal. As was shown, even small errors severely affect the correlator-based detector performance. To solve this problem, an NN-based detector was proposed. Such a detector highly outperforms traditional FSK correlator-based demodulator. The additional use of channel coding improves the performance of the system. In this case, the metrics calculated based on NN outperformed the theoretically calculated metrics for the correlator-based detector. It leads to better soft-decision decoding. Ultimately, the simulation results showed that the NN-base demodulator with the Viterbi decoder provides error-free demodulation, even for normalized channel gain estimator error$\;e_{abs}$ = 2%, compared to $e_{abs}$ = 0.5% for the correlator-based demodulator. Because of focusing on non-ideal SIC operation, the impact of channel noise, multipath transmission, and additional radiated undesirable missions [29] were omitted here for clarity. This may be a subject of future studies.

## Figures and Tables

**Table 1:** Parameters of the simulation.

Modulation	Variance	Number Samples Per Symbol
16QAM (cover)	$\sigma_{1}^{2}$	1
BPSK (covert)	$0.05\cdot \sigma_{1}^{2}$	8
2FSK (covert)	$0.05\cdot \sigma_{1}^{2}$	8
8FSK (covert)	$0.05\cdot \sigma_{1}^{2}$	24

**Table 2:** Simulation parameters.

Signal	Modulation	Bit Rate	Variance	Signal
cover	16QAM	9600bps	$\sigma_{1}^{2}$ = 1	cover
covert	8FSK	600bps	$\sigma_{2}^{2}$ $\;=\;0.01\sigma_{1}^{2}$	covert

## Data Availability

The data presented in this study are available on request from the corresponding author. The data are not publicly available due to project restrictions.
